# Social Listening: A Content Analysis of E-Cigarette Discussions on Twitter

**DOI:** 10.2196/jmir.4969

**Published:** 2015-10-27

**Authors:** Heather Cole-Lewis, Jillian Pugatch, Amy Sanders, Arun Varghese, Susana Posada, Christopher Yun, Mary Schwarz, Erik Augustson

**Affiliations:** ^1^ ICF International Rockville, MD United States; ^2^ National Cancer Institute Tobacco Control Research Branch Bethesda, MD United States

**Keywords:** social media, Twitter, e-cigarettes, content analysis

## Abstract

**Background:**

Electronic cigarette (e-cigarette) use has increased in the United States, leading to active debate in the public health sphere regarding e-cigarette use and regulation. To better understand trends in e-cigarette attitudes and behaviors, public health and communication professionals can turn to the dialogue taking place on popular social media platforms such as Twitter.

**Objective:**

The objective of this study was to conduct a content analysis to identify key conversation trends and patterns over time using historical Twitter data.

**Methods:**

A 5-category content analysis was conducted on a random sample of tweets chosen from all publicly available tweets sent between May 1, 2013, and April 30, 2014, that matched strategic keywords related to e-cigarettes. Relevant tweets were isolated from the random sample of approximately 10,000 tweets and classified according to sentiment, user description, genre, and theme. Descriptive analyses including univariate and bivariate associations, as well as correlation analyses were performed on all categories in order to identify patterns and trends.

**Results:**

The analysis revealed an increase in e-cigarette–related tweets from May 2013 through April 2014, with tweets generally being positive; 71% of the sample tweets were classified as having a positive sentiment. The top two user categories were everyday people (65%) and individuals who are part of the e-cigarette community movement (16%). These two user groups were responsible for a majority of informational (79%) and news tweets (75%), compared to reputable news sources and foundations or organizations, which combined provided 5% of informational tweets and 12% of news tweets. Personal opinion (28%), marketing (21%), and first person e-cigarette use or intent (20%) were the three most common genres of tweets, which tended to have a positive sentiment. Marketing was the most common theme (26%), and policy and government was the second most common theme (20%), with 86% of these tweets coming from everyday people and the e-cigarette community movement combined, compared to 5% of policy and government tweets coming from government, reputable news sources, and foundations or organizations combined.

**Conclusions:**

Everyday people and the e-cigarette community are dominant forces across several genres and themes, warranting continued monitoring to understand trends and their implications regarding public opinion, e-cigarette use, and smoking cessation. Analyzing social media trends is a meaningful way to inform public health practitioners of current sentiments regarding e-cigarettes, and this study contributes a replicable methodology.

## Introduction

In recent years, electronic cigarette (e-cigarette) use has gained momentum in the United States, with 36.5% of current smokers surveyed in 2013 reporting ever using e-cigarettes, compared to 9.8% in 2010 [1]. From 2010 to 2013, e-cigarette awareness increased nearly 40%, and “ever use” of e-cigarettes increased in all demographic subpopulations except those aged 18-24 years, Hispanics, and those living in the Midwest of the United States. Results from the National Youth Tobacco Survey indicate that current e-cigarette use among high-school students tripled from the previous year, which marks the first time that current e-cigarette use has surpassed current use of every other tobacco product [2]. Data from the recent cycle of the Health Information National Trends Survey (HINTS 4 Cycle 2) indicate that among people aware of e-cigarettes, 51% believe e-cigarettes are less harmful than conventional cigarettes [3]. The growing popularity of e-cigarettes within the United States and worldwide has resulted in a surge of research spanning topics such as harm reduction, use patterns (cessation vs dual use), health effects, environmental effects, marketing, and product design. To date, data providing guidance to public health decision makers is still being collected and there is an active debate within the public health sphere regarding e-cigarette use and regulation [4]. Current findings and opinions range considerably. While some believe e-cigarettes are helping to end the global morbidity and mortality associated with the use of combustible tobacco, others believe that e-cigarettes threaten to prolong or worsen the tobacco epidemic [4-8]. Although these stances are part of the ongoing debate, there is general support for the regulation of e-cigarettes. Experts within the United States have urged the US Food and Drug Administration (FDA) to take the necessary actions to bring e-cigarettes under their regulatory authority [9], and the FDA has signaled their intention to do so [10]. However, data collection that fully addresses the public health impact of e-cigarettes and the implementation of e-cigarette regulation may take some time. In the meantime, e-cigarette sales are climbing rapidly. E-cigarette retail sales in 2013 were approximately US $2.5 billion worldwide. With this current trajectory, it is projected that retail sales in 2017 will top US $10 billion [11,12].

Given that e-cigarettes are still relatively new and the opinions toward them are often divergent, there is increasing dialogue surrounding e-cigarettes on social media. As we aim to understand the health effects of e-cigarettes, we must also attempt to discern the core voices, message frames, and sentiment surrounding e-cigarette discussions. Understanding these conversations allows public health and communication professionals to identify trends in attitudes and behaviors and to develop strategies to disseminate factual information and create culturally relevant cessation interventions for nicotine products, including traditional cigarettes and e-cigarettes.

Analysis of Twitter data has become an active research area, offering insight for the behavioral and social sciences and providing access to demographic groups that are often underrepresented in research, such as minorities. According to the Pew Research Center [13], Twitter usage is particularly popular among “younger adults, urban dwellers, and non-whites.” Twitter offers greater representation of minority groups with 25% of Latinos, 27% of blacks, and 21% of whites using the social media platform [14]. In addition, 31% of 18-29-year olds use Twitter, as compared to 18% of all adult Internet users [13]. The microblog format acts as a social support system for sharing information, ideas, and beliefs that can be captured in real-time, offering insight that survey analyses might miss or take an extended period to collect [15].

In the case of tobacco use and cessation, examination of social media data can continue to uncover trends in knowledge, attitudes, and behavior; identify marketing strategies; inform public health and public policy; and pave the way for interventions delivered via social media [16-22]. Use of social media information to detect public health trends in this way has been referred to as “infoveillance” or “infodemiology” [23-26], and as digital epidemiology or digital disease detection [27].

Given the depth of data, the breadth of its audience, and its ability to capture real-time trends, this study focuses exclusively on understanding snapshots of dialogue surrounding e-cigarettes captured on Twitter. The objective of this analysis is to conduct a content analysis to identify key conversation trends and patterns over time using historical Twitter data.

## Methods

To conduct this analysis, we used strategic keywords to collect historical tweets potentially related to e-cigarettes from May 1, 2013, to May 1, 2014. Keywords were selected using an iterative process with incremental addition and subtraction of words. A preliminary search, using words like e-cigarette and vapor was conducted, followed by refinement to remove terms capturing tweets that were not relevant. Addition of words was heavily influenced by a list of previously published keywords [22]. We ran several sample searches using Radian 6, social media insight software, and informally tested the performance of the searches using frequencies, correlation tables, and descriptive visualizations such as bar graphs. Product names were omitted from search keywords because we were unable to identify an exhaustive list of every e-cigarette product name and did not want to bias results to specific brands. The final list of strategic keywords is provided in Multimedia Appendix 1.

Data were provided by Gnip, a company with full access to the Twitter Firehose (entire stream of Twitter data) supplying historical tweets not available through the Twitter application program interface (API). Search results garnered 3.7 million potentially relevant tweets. Gnip data utilized for the purposes of this study include time, date, user profile link, tweet content, and tweet link. To facilitate user-friendly evaluation of the tweets among 6 analysts, a database and Web form were developed that prepopulated each tweet along with the coding categories (see Multimedia Appendices 2 and 3). Each tweet included a link to the Twitter post and the user profile. In instances where the tweet link had been removed, analysts used a historic Web cache to capture the information. Analysts visited full webpage and user profiles at their discretion when sufficient information about the tweet could not be obtained from the extraction. We did not track the number of webpage and user profile clicks made by analysts, but anecdotally, analysts clicked these links for approximately half of all coded tweets. All analysts were college educated and participated in training sessions to familiarize themselves with e-cigarette topics and tweet analysis techniques with the lead author prior to analyzing the tweets.

Manual content analysis was used to categorize tweets according to a coding category list developed through previous literature and adapted for the purposes of this research [22]. The content analysis consisted of two stages: (1) randomly sampling tweets from the full dataset and classifying content for e-cigarette relevance until a manageable sample of at least 10,000 relevant tweets was achieved and (2) classifying content of each relevant tweet for sentiment, user description, genre, and theme. Each stage consisted of an initial step wherein analysts coded 250 of the same tweets until an acceptable level of interrater agreement was reached. Interrater reliability was determined using the Fleiss kappa and a score of at least .64 was obtained for each category in Stages 1 and 2, indicating substantial or good agreement [28,29]. Classification for relevance during Stage 1 excluded tweets that met any of the following specifications: “retweets” that offered no additional information from the person posting the tweet, original tweets that were part of a conversation and require greater context to be interpreted, or duplicated tweets from a user account that had since been suspended or was primarily being used for spam or unwanted solicitations. Spam was identified according to guidelines outlined by Twitter and included consideration of factors such as updates that are mostly links and not personal updates, duplicate content posted over multiple accounts, and content that consists of unrelated hashtags of popular topics [30,31].

Classification for sentiment, user description, genre, and theme in Stage 2 was conducted according to a codebook developed for the classification of tweets that builds on previous research [17,22] and the focus of this analysis (see Multimedia Appendix 3). Sample tweets for each content category are included in Multimedia Appendix 4).

*Sentiment* refers to whether the stance in the tweet is positive, neutral, or negative toward e-cigarettes and users of e-cigarettes (Table 1). Stance toward e-cigarettes and users of e-cigarettes were the only considerations for sentiment, and sentiment toward any other topic or concept was disregarded for the purposes of this research.

**Table 1:** Content categories for sentiment.

Category	Definition
Positive	Tweets that are in favor of e-cigarettes, related products, and use
Neutral	Tweets not strong in either direction for or against e-cigarettes
Negative	Tweets with that are against e-cigarettes

*User description* characterizes the sender of the tweet based on information gleaned from the user profile (eg, e-cigarette company, everyday user of Twitter, reputable news source; see Table 2). In particular, the user profile category of “e-cig community movement” was added specifically to represent those people who appear to be strong advocates for e-cigarettes but have no identified affiliation with marketing or e-cigarette companies. This category is distinct from the “everyday person” category in that the great majority of the user’s timeline is devoted to e-cigarette advocacy and information, with little mention of the activities of day-to-day life characteristic of those who use Twitter for personal purposes.

**Table 2:** Content categories for user description.

Category	Definition
Celebrity	Famous people in pop culture, people that are Internet famous, people that have accounts verified by Twitter
Government	National Institutes of Health, CDC, political figures, etc
Foundation/organization	Reputable organizations such as American Heart Association
Reputable news source	New sources such as New York Times, Washington Post, Wall Street Journal, Associated Press, etc
Everyday person	Twitter account with a reasonable amount of posts, followers, and following a reasonable amount of people; timelines span a variety of topics that are not primarily e-cigarette–related
E-cigarette community movement	Groups or people whose timelines are primarily devoted to e-cigarette conversation (eg, Women Who Vape, The Vape Club, John Doe with entire timeline of e-cigarette tweets)
Retailer	Outlets that sell e-cigarettes (online or physical)
Tobacco company	Companies that manufacture e-cigarettes (eg, blu, Apollo, Njoy)
Bot/hacked	Accounts that appear to be fake/computerized that are primarily promoting e-cigarette products (or other products); most accounts are disguised to appear as “everyday person”

*Genre* represents the format of the tweet (eg, news or update, first person experience, marketing; see Table 3). *Theme*, the most granular level of classification, refers to the topical domain of the content in the tweet (eg, cessation, health and safety, craving; see Table 4).

**Table 3:** Content categories for genre.

Category	Definition
News/update	Update about a current event from a reputable news source, or post from user about relevant news from news source
Information	Factoid or resource, can be a personal blog or forum, or link to product review (posted by everyday person or e-cigarette community movement)
First person e-cigarette use or intent	Reports personal use of, intent, or interest to use e-cigarettes
Second/third person experience	Reports someone else’s use of e-cigarette
Personal opinion	Personal opinion related to e-cigarettes
Marketing	Activities involved in the transfer of goods from the producer or seller to the consumer or buyer, eg, sales of e-cigarette products or accessories, job announcements, review of products posted by e-cigarette company/retailer

**Table 4:** Content categories for theme.

Category	Definition
Cessation	Mention of using e-cigarettes to quit smoking cigarettes or other non-e-cigarette tobacco products
Health and safety	Direct or indirect reference to health consequences of e-cigarette use
Underage usage	E-cig use by minors, especially high-school age or under
Craving	Desire to use e-cigarettes; eg “Stressful day. Time for my #vapepen”
Other substances	E-cigarettes mentioned in association with other addictive substances (eg, alcohol, caffeine)
Illicit substance use in e-cigarettes	Mention of using e-cigarettes for anything other than nicotine (eg, marijuana)
Policy or government	Mention of government or policy in relation to e-cigarettes including regulation, deeming, bans, and restrictions
Parental use of e-cigarettes	Tweet mentioning use of e-cigarettes by parents of the poster or parents of a person mentioned in the tweet
Advertisement/ promotion	Ads for e-cigarettes, giveaways, samples, sales, direct links to sellers’ websites, word-of-mouth, and reviews
Flavors	Tweet discussing e-cigarette flavors (generic or mixed, including menthol)

Sentiment and user description are mutually exclusive categories—meaning that only one choice could be made per category, while genre and theme are not—meaning that more than one choice could be made per category. All categories were mandatory with the exception of theme, given the granularity of the content and because every topic could not be realistically represented. Additionally, during Stage 2, analysts documented media links included in each tweet (eg, image, video, location, website).

After the content analysis was complete, descriptive statistical analyses were performed on the data sample, including one-way frequencies for each category; two-way cross tabulations for categories, temporal trends, and media type, in addition to the chi-square test for intercategory statistical association (using Fisher’s exact test for cell counts ˂5); and intercategory correlation analysis based on Cramer’s V coefficient (representing each category option as a binary variable). Both the chi-square tests and correlation analyses with Cramer’s V provide a statistically sound assessment of the significance and strength of the relationships between various categories. SAS version 9.3 was used for all analyses. The goal of the current analysis was to identify patterns and trends in the sample of tweets related to the overarching content categories: sentiment, user description, genre, and theme.

General trends are reported for the entire sample of coded tweets; only statistically significant trends are discussed for each category (*P*<.05). Additionally, intercategory trends are reported, once again discussing only the principal statistically significant findings (*P*<.05) of interest based on bivariate associations and intercategory correlation assessments.

## Results

### Sample Description

A total of 17,098 tweets were coded during Stage 1, of which 10,128 (59.23%) were found to be relevant and interpretable. The range of interrater reliability was .64-.70 and is reported in Table 5. Of the excluded tweets, 2384 were found to be entirely non-relevant, whereas the remainder were retweets with no additional context, conversations without context, or duplicated tweets from a user account that had since been suspended or was primarily being used for spam or unwanted solicitations. For the remainder of this discussion, the final sample consisted of the 10,128 relevant tweets.

**Table 5:** Interrater reliability scores for manual annotation of tweet categories.

Category	Interrater reliability
Relevance^a^	.70
Sentiment^b^	.65
User description^b^	.66
Genre	.64
Theme	.65

^a^Binary version of this category was created in addition to multiclass version for the purposes of the analysis.

^b^Categories were mutually exclusive and thus analyzed as multiclass.

**Figure 1:**
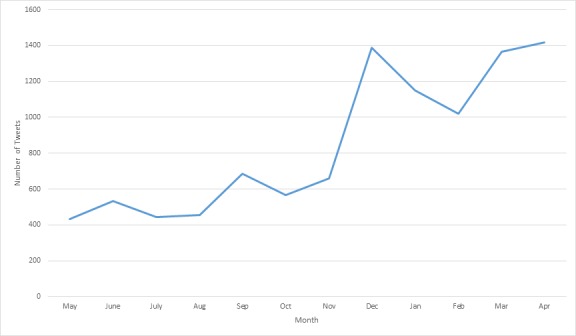
Frequency of e-cigarette tweets by month from May 2013 to April 2014.

Between May 2013 and November 2013, each month contributed 4.29-6.53% of the tweets in the overall sample; however, there is a clear increase in the number of relevant e-cigarette tweets in December 2013. The number of tweets in December 2013 (n=1388) is more than twice the number of tweets that occurred in November 2013 (n=631; see Figure 1). Months between December 2013 and April 2014 each represent 10.09% to 14.01% of the total tweets in the sample, which represents a bulk of the tweets that occurred during the observation period (see Figure 1).

Almost half of the tweets (48.00%) included links that were functional at the time of the content analysis. Tweets with images accounted for 8.30% of the sample.

### Sentiment

**Figure 2:**
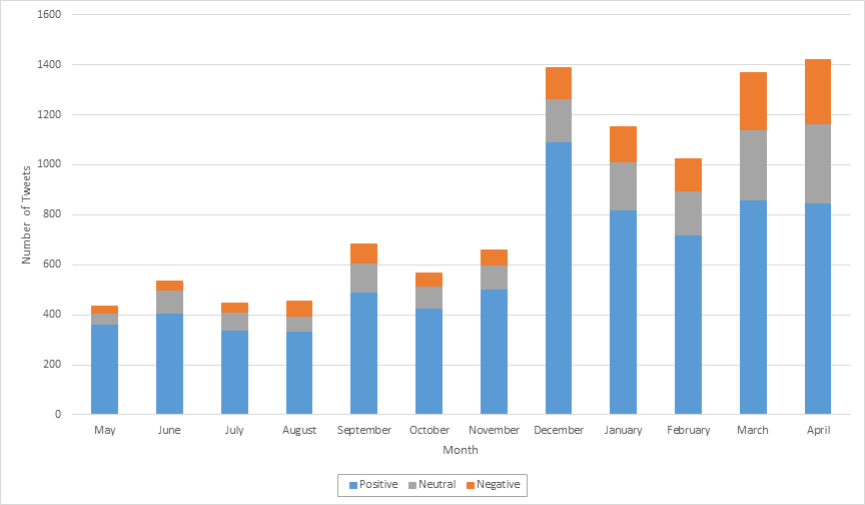
Absolute number of tweets by sentiment and month from May 2013 to April 2014.

As indicated by Table 6, tweets deemed positive in sentiment accounted for a majority of the sample (71.11%). The absolute number of positive tweets was highest in December 2013, but May 2013 had the highest percentage of positive sentiment tweets (Figure 2). There was a steady decline in positive sentiment from December 2013 through April 2014, during which the percentage of negative and neutral tweets rose. This resulted in April 2014 having the highest percentage of negative (17.90%) and neutral tweets (22.48%).

**Table 6:** Tweet distribution by sentiment (N=10,128).

Sentiment	N (%)
Positive	7202 (71.11)
Neutral	1699 (16.78)
Negative	1227 (12.11)

### User Description

**Figure 3:**
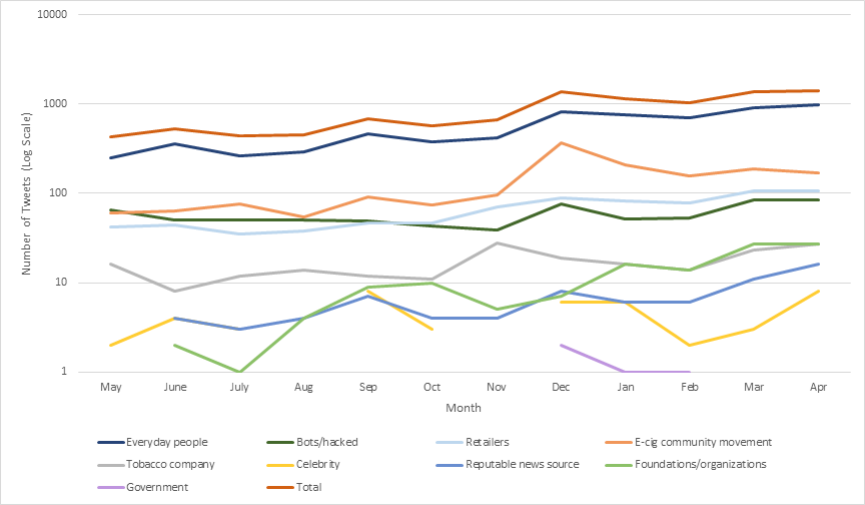
Number of tweets by month and user from May 2013 to April 2014.

A majority of the sample consisted of tweets that originated from users identified by analysts as everyday people (64.99%), with the second largest population being the e-cigarette community (15.92%) (see Table 7). Tweets originating from government, celebrity, and reputable news sources user accounts each represented less than 1% of the sample. November 2013 saw the highest percentage of tweets from retailers (10.59%) and tobacco companies (4.24%), while the proportion of tweets from e-cigarette community movement users peaked in December 2013 (26.72%) (see Figure 3).

**Table 7:** Tweet distribution by user description (N=10,128).

User description	N (%)
Celebrity	45 (0.44)
Government	8 (0.08)
Foundations/organization	122 (1.20)
Reputable news source	73 (0.72)
Everyday person	6582 (64.99)
E-cigarette community movement	1612 (15.92)
Retailer	787 (7.77)
Tobacco company	200 (1.97)
Bot/hacked	699 (6.90)

### Genre

The three most common tweet genres were personal opinion-oriented tweets, marketing-related tweets, and personal experience-related tweets (see Table 8). The monthly volume of personal opinion-related tweets more than doubled during the analysis period, with a marked increase occurring from October 2013 to a peak in March 2014, though the percentage of these tweets was highest in February 2014 (see Table 9). Marketing-related tweets saw a fairly steady decline between May 2013 (29.95%) and April 2014 (18.46%) although the absolute number of these tweets doubled in that period. Personal opinion-related tweets accounted for more than one-fifth of each month’s tweets, with a spike in volume occurring in December 2013, which accounted for nearly half of that months’ e-cigarette related tweets (44.52%). The percentage of news-related tweets increased from 1.84% to 15.22% from May 2013 to April 2014, which represents a 27-fold increase in the volume of news-related tweets during that time. Marketing, news, and information-related tweets have much higher rates of website links (60.12%, 88.51%, and 70.40%) than average (35.42%).

**Table 8:** Tweet distribution by genre (N=10,128).

Genre	N (%)
News/update	828 (8.18)
Information	1459 (14.41)
First person e-cigarette use or intent	2056 (20.30)
Second/Third person experience	797 (7.87)
Personal opinion	2850 (28.14)
Marketing	2142 (21.15)

**Table 9:** Tweet genre distribution by month (N=10,128).

Genre	N (%)												
	May	June	July	Aug.	Sept.	Oct.	Nov.	Dec.	Jan.	Feb.	Mar.	Apr.	Total
Personal experience	102 (23.50)	122 (22.90)	109 (24.49)	106 (23.35)	131 (19.12)	134 (23.63)	152 (23.00)	196 (14.12)	230 (19.97)	248 (24.27)	270 (19.74)	256 (18.04)	2056
Marketing	130 (29.95)	140 (26.27)	128 (28.76)	115 (25.33)	157 (22.92)	130 (22.93)	167 (25.26)	239 (17.22)	225 (19.53)	174 (17.03)	272 (19.88)	262 (18.46)	2139
Personal opinion	95 (21.89)	112 (21.01)	97 (21.80)	105 (23.13)	191 (27.88)	137 (24.16)	176 (26.63)	618 (44.52)	363 (31.51)	285 (27.89)	349 (25.51)	321 (22.62)	2849
Second person	34 (7.83)	52 (9.76)	34 (7.64)	45 (9.91)	50 (7.30)	37 (6.53)	48 (7.26)	82 (5.91)	96 (8.33)	93 (9.10)	112 (8.19)	114 (8.00)	797
Information	65 (15.00)	74 (13.88)	51 (11.46)	62 (13.66)	99 (14.45)	93 (16.40)	82 (12.41)	165 (11.89)	150 (13.02)	145 (14.19)	221 (16.15)	249 (17.55)	1456
News	8 (1.84)	33 (6.19)	26 (5.84)	21 (4.63)	57 (8.32)	36 (6.35)	35 (5.30)	87 (6.27)	88 (7.64)	77 (7.53)	143 (10.45)	216 (15.22)	827
Total	434	533	445	454	685	567	661	1388	1152	1022	1368	1419	10,128

### Theme

Table 10 describes the overall themes present in the dataset. During the coding process, tweet theme was not a mutually exclusive category, and this resulted in 26.35% of tweets in the sample having more than one theme. For tweets with one theme, advertising and promotions-related tweets were the single largest content theme category with 19.62% occurrence in the sample, followed by policy and government–related tweets at 11.77% and health and safety–related tweets at 4.27%. Tweets coded for only the cessation theme accounted for 1.42% of the sample.

**Table 10:** Tweet distribution by theme.^a^

Theme	N (%)
Cessation	638 (6.30)
Health and safety	1327 (13.10)
Underage usage	423 (4.18)
Craving	394 (3.89)
Other substances	116 (1.15)
Illicit substance use in e-cigarettes	160 (1.58)
Policy/government	2042 (20.16)
Parental use of e-cigarettes	74 (0.73)
Advertisement/promotion	2663 (26.29)
Flavors	451 (4.45)

^a^Includes tweets coded with multiple themes.

### Intercategory Trends

#### Bivariate Associations

The bivariate associations reported are statistically significant (*P*<.05). Almost all marketing-related tweets were positive in sentiment (98.46%), while 88.72% of first person e-cigarette use or intent and 69.78% of personal opinion tweets were positive. Approximately half (51.65%) of informational tweets were positive and 14.22% were negative. News-related tweets were the least positive of the genres, with 19.11% of these tweets coded as positive, 53.10% neutral, and 27.81% negative.

Over 92.27% of tweets containing an image were positive in sentiment. Retailers accounted for 19.74% of tweets containing images and marketing-related tweets are twice as likely to contain an image (17.30%) compared to the average rate at which images occur in tweets (8.30%). E-cigarette community users produced 23.47% of tweets containing a link to a website. Marketing, news, and information-related tweets have much higher rates of website links (60.12%, 70.40%, and 88.51%) than the overall average (35.43%).

**Figure 4:**
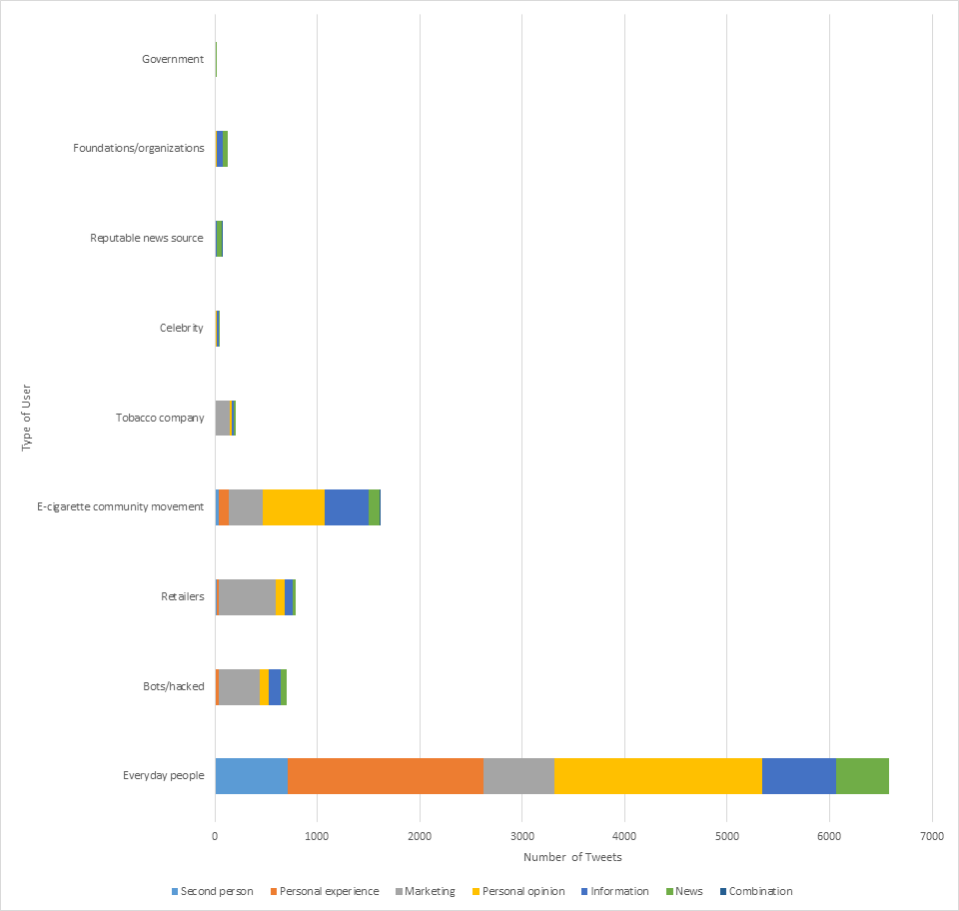
Distribution of tweet content genre for each user category.

Nearly half (49.60%) of information tweets originated from everyday people and 29.28% were from e-cigarettes community movements. Everyday people represented 62.27% of news tweets as compared to reputable news sources accounted for 6.41% of news and 0.96% of information tweets. Foundations/organizations provided 3.98% of information tweets and 5.20% of news tweets. Also, 32.40% of marketing tweets came from everyday people compared to 26.18% from retailers and 6.40% from tobacco companies. User-related trends in tweet genre are illustrated in Figure 4.

#### Correlation Analysis

As an additional measure of correlation, Cramer’s V statistic was computed for all categories after representing each category option as a binary variable. Multimedia Appendix 5 displays the results of the Cramer’s V correlation analysis, highlighting those correlations that are moderately strong (≥.3). Results are similar to the bivariate associations discussed previously.

## Discussion

### Principal Findings

This content analysis revealed noteworthy trends about e-cigarette–related tweets from May 2013 through April 2014. The number of these tweets rose during the data collection period, with a peak in December 2013. Tweets were overwhelmingly positive and frequently posted by everyday people and e-cigarette community movement accounts.

The increase in e-cigarette-related tweets coincides with several e-cigarette milestones, ranging from government proposals for policies and regulations [32], major tobacco corporations introducing their own brands of e-cigarettes and buying of existing ones [33], and the e-cigarette industry’s increased visibility through marketing and retail expansion. This trend may also have been driven by e-cigarette promotional activities and the subsequent increase in sales and awareness of e-cigarette products [34]. For instance, in February 2014 the company NJOY King ran an e-cigarette advertisement during the Super Bowl for a second consecutive year [35]. This televised event was viewed by over 111 million people in America, making it the most-watched television event in history at the time [36].

From May 2013 through April 2014, everyday people dominated the e-cigarette conversation on Twitter by accounting for over two-thirds of the tweets in the dataset. The e-cigarette community movement represented the second most common user type. As expected, everyday people accounted for the majority of personal opinion and personal experience tweets, though e-cigarette community movement accounts represented a sizeable proportion of personal opinion tweets as well. These two user groups accounted for 80% of information tweets, with minimal information coming from government, public health non-governmental organizations, and reputable news sources. Future research may look into the legitimacy of the information shared, its origins, and how it is shared across Twitter. This will help us understand the degree to which e-cigarette information spreads and how that might impact beliefs and opinions surrounding e-cigarettes. Everyday people also tweeted nearly a third of the marketing-related tweets, which is equivalent to the percentage of marketing tweets from retailers and tobacco companies combined. It is important to note that the volume of tweets from a particular user group does not reflect the reach or number of impressions their tweets made as this analysis did not take into account the number of followers a Twitter account has, nor the number of retweets or favorites a tweet received. Although this is a limitation of the current study, it presents the opportunity for future research to determine which Twitter voices are the “loudest” in the sense that their tweets are being seen and shared most often, and how these visible tweets influence perceptions and use of e-cigarettes.

E-cigarette community tweets spiked in December 2013, which represents a four-fold increase in tweet volume from the prior month. The cause of the sharp rise in e-cigarette community movement tweets remains unknown, but there were several e-cigarette milestones during this time. For example, in December 2013 Phillip Morris International Inc. announced its partnership with Altria Group Inc. to sell e-cigarettes [37]. A quarter of e-cigarette community tweets contained a Web link, which was considerably higher than the average. This is worth mentioning because Web links are often marketing vehicles, with 60% of the marketing tweets in this dataset containing Web links. The use of Web links among e-cigarette community users may indicate consumers’ willingness to drive marketing efforts and contribute to the normalization and popularization of e-cigarettes.

Tweets originating from reputable news sources and government agencies comprised less than 1% of the sample. There continues to be debate regarding how to regulate e-cigarettes within the United States and many countries. Our analysis from Twitter suggests that the uncertainty expressed within the field of public health is not reflected in the nature of the ongoing social media dialogues. In the absence of informative dialogue from public health authorities, personal opinion and marketing content surrounding e-cigarettes have become the most common themes. This sample shows a decisive dip in tweets originating from accounts that were clearly marketers of e-cigarette products, but a large amount of marketing content continues to be posted by individuals and e-cigarette communities.

In addition to understanding who is talking on Twitter, it is necessary to dissect what is being said. Most tweets were determined to have positive sentiment indicating that Twitter dialogue skews favorably toward e-cigarettes, although the proportion of positive tweets declined during the analysis period. This trend warrants further monitoring with specific consideration for what fuels opinion over time. Furthermore, this research establishes an e-cigarette sentiment baseline that serves as a valuable starting point for public health professionals to develop campaigns and interventions. A majority of marketing-related tweets had positive sentiment, while approximately one-fifth of news-related tweets were positive. The most prevalent genre uncovered was personal opinion, followed closely by marketing to comprise nearly half of the sample. It can be expected that a platform like Twitter is conducive to sharing personal opinion; however, 32% of marketing tweets came from everyday people, while 32% came from retailers and tobacco companies combined.

### Strengths and Limitations

As with any research study, our study has limitations. It must be noted that our analysis is quite specific to the topic of e-cigarettes, and thus our keyword list was limited to terms directly related to e-cigarettes. It is possible that we have overlooked conversations around topics that are socially similar to, but not exactly the same as e-cigarettes such as e-hookah. In addition, the vocabulary surrounding e-cigarettes is continuously growing and changing. This is due to the expanding range of products, brands, and vaping-related activities that people engage in. As a result, the list of keywords used in this study would need to be reconsidered and almost certainly expanded to accommodate the changing e-cigarette and vaping terminology. Furthermore, calculation of precision and recall for the search would have provided a better understanding of the validity of terms retrieved by our search. However, we are confident that our methodology is replicable, with appropriate resources and thus would allow for expansion in order to explore other emerging trends. We recommend calculation of precision and recall to refine the search and report validity using a systematic quantitative method such as that described by Stryker et al [38].

Additionally, our exclusion methodology for relevance, which included eliminating retweets without additional information and duplicate tweets from suspended accounts, may have led to an underestimation of the true prevalence of these types of tweets. However, we believe that even if our study provides a conservative estimate of the information available on Twitter in relation to e-cigarettes, the information remains useful to gain an understanding of the general trends. Future studies may be interested in utilizing less restrictive relevance criteria and using methods such as social network analysis to determine the structure of the network and how this relates to dissemination of e-cigarette attitudes and perspectives.

Twitter users do not represent the general population, and thus findings from this study must be considered in the context of people who use this specific social media platform [33]. Nonetheless, given the popularity of Twitter especially among youth, black, and Latino populations [14], information from studies such as ours provides an opportunity to access public opinions from this particular subset of the population and use this information to form hypotheses and inform future research, as well as to supplement prior research.

Additionally, in our analysis, we did not apply an explicit weighting or correction methodology to adjust for changes in tweet volumes over time because any approach to making such an adjustment would potentially bias results. Given that most of our descriptive metrics compare fractions of tweets of a specific classification across points in time rather than absolute numbers of tweets, we believe that the comparative picture presented of the e-cig–related tweet landscape as it evolves over time is valid.

Despite a few limitations, there were many strengths of this analysis. Our study accessed data from the Twitter Firehose (ie, access to all of the daily tweets on Twitter) and utilized a large sample of tweets. Moreover, analyses were carried out over a critical period of time in the e-cigarette landscape and expanded on previously established methodologies for thematic analysis of Twitter.

An additional key strength of this work is the significant amount of time and effort spent manually building a dataset that is sampled from the Twitter Firehose (rather than the free API). The dataset of 10,128 manually coded tweets for this study is a much larger sample than previous work on Twitter and emerging tobacco products [21,22]. For example, the study presented by Huang et al included 2000 manually coded tweets, which served as the machine learning training set for over 75,000 e-cigarette-related tweets. Myslín et al’s work greatly influenced our study, though it included traditional tobacco products and other emerging products (eg, hookah) in addition to e-cigarettes [22]. Myslín et al’s study used over 7300 manually coded tweets for its machine learning training set, with only 4200 being relevant to tobacco, and fewer than 100 of these tweets were related to e-cigarettes.

For future research, the data from this content analysis can be used as a training dataset to build supervised machine learning algorithms. These algorithms can be used to implement automated surveillance of e-cigarette-related conversations on Twitter. This would allow more data to be analyzed with less manpower and also allow observation and analysis of Twitter trends for e-cigarette conversations over a greater period of time. This form of infoveillance lends itself to several aspects of tobacco control, including marketing regulations, underage use, cessation, and health outcomes.

### Conclusion

Continuing snapshots of the social media landscape around e-cigarettes may help policymakers and public health professionals assess changing trends and inform interventions for tobacco cessation. Identifying means to integrate these types of assessments and analyses into data collected by traditional epidemiology and surveillance methods may prove especially valuable [22]. Also, this study highlights a replicable methodology and 5-category coding scheme that could be used in the future for additional topic areas.

## Appendix

Multimedia Appendix 1 Tweet filter keywords.

Multimedia Appendix 2 Data collection Web form.

Multimedia Appendix 3 Definitions of annotation categories.

Multimedia Appendix 4 Sample tweets by annotation category.

Multimedia Appendix 5 Correlation matrix for content categories.

## Abbreviations

API: Application Program Interface
CDC: Centers for Disease Control and Prevention
FDA: Food and Drug Administration
HINTS: Health Information National Trends Survey
SAS: Statistical Analysis System
