# Supplementary material for: Social Listening: A Content Analysis of E-Cigarette Discussions on Twitter
Source: J Med Internet Res. 2015 Oct 27;17(10):e243. doi: 10.2196/jmir.4969 (PMC4642379; doi:10.2196/jmir.4969)
Supplement: Multimedia Appendix 2 [file jmir_v17i10e243_app2.pdf]

Multimedia Appendix 2. Data collection web form.

387) Final testing phase of new flavour can't wait to get this out to everyone #vape #vaping #ejuice #eliquid #vapelife http://t.co/[redacted]
Author: [redacted]
Posted Date: Thu Apr 16 14:14:57 +0000 2015

User Description

- ( ) Celebrity
- ( ) Government
- ( ) Foundations or organizations
- ( ) Reputable News Source
- ( ) Every day people
- ( ) E-cig community movement
- ( ) Retailers
- (•) Tobacco Company
- ( ) Bots/Hacked

Sentiment

- ( ) Negative
- ( ) Neutral
- (•) Positive

Content Type A

- [ ] News/update
- [ ] Info
- [ ] 1st person e-cig use or intent
- [ ] Second/Third person experience
- [ ] Personal opinion
- [x] Marketing

Content Type B

- [ ] Link to an image
- [x] Link to a video
- [ ] Link to a location
- [ ] Lint to a website
- [ ] Broken Link
- [ ] No Link

Content Theme

- [ ] Cessations
- [ ] Emotional Response
- [ ] Illicit substance use in e-cigs
- [ ] Normalization of e-cigs
- [ ] Health and Safety
- [ ] Craving
- [ ] Policy or Government
- [x] Advertisement/ Promotion
- [ ] Underage usage
- [ ] Other substances
- [ ] Parental use of e-cigs
- [x] Flavors

Comments

Save
