# Supplementary material for: Social Listening: A Content Analysis of E-Cigarette Discussions on Twitter
Source: J Med Internet Res. 2015 Oct 27;17(10):e243. doi: 10.2196/jmir.4969 (PMC4642379; doi:10.2196/jmir.4969)
Supplement: Multimedia Appendix 3 [file jmir_v17i10e243_app3.pdf]

Multimedia Appendix 3. Definitions of annotation categories.

Relevance: Identifies tweets that are related to e-cigarettes.	
Relevant	Tweet that includes topics related to e-cigarettes. Subcategories include: retweets that offered no additional information from the person posting the tweet; original tweets that were part of a conversation and require greater context to be interpreted; or duplicated tweets from a user account that had since been suspended or was primarily being used for spam or unwanted solicitations.
Not relevant	Tweets that do not include information related to e-cigarettes.
Sentiment: Indicates whether the stance in the tweet is positive, neutral, or negative towards e-cigarettes and users of e-cigarettes.	
Positive	Tweets that are in favor of e-cigarettes (eg, like, happy, uses words like "genius" or #lovevape; boasting about e-cigarette use).
Neutral	Tweets not strong in either direction for or against e-cigarettes.
Negative	Tweets with that are against e-cigarettes (eg, don't like, not impressed, uses words like "hate" or #EPICFAIL).
User description: Characterizes the sender of the tweet based on information gleaned from the user profile (eg, e-cigarette company, everyday user of Twitter, reputable news source).	
Celebrity	Famous people in pop culture, people that are internet famous, people that have accounts verified by Twitter.
Government	National Institutes of Health, Centers for Disease Control and Prevention, Political figures, etc.
Foundations or organizations	Reputable organizations such as American Heart Association.
Reputable news source	New sources such as New York Times, Washington Post, Wall Street Journal, Associated Press, etc.
Everyday people	Twitter account with a reasonable amount of posts, followers, and following a reasonable amount of people with a timeline that spans a variety of topics that are not primarily e-cigarette related.
E-cigarette community movement	Groups or person whose timelines are primarily devoted to e-cigarette conversations (eg, Women Who Vape, The Vape Club, John Doe with entire timeline of e-cigarette tweets).
Retailers	Outlets that sell e-cigarettes (online or physical).
Tobacco company	Companies that manufacture e-cigarettes (eg, blu, Apollo, Njoy).
Bots/hacked	Accounts that appear to be fake/computerized that are primarily promoting e-cigarette products (or other products); most accounts are disguised to appear as "everyday users."
Genre: Represents the format of the tweet (eg, news or update, first person experience, marketing).	
News/update	Update about a current event from a reputable news source, or post from user about relevant news from news source.
Information	Factoid or resource, can be a personal blog or forum, or link to product review (posted by every day user or e-cigarette comm. Movement).

First person e-cigarette use or intent	Reports personal use of, intent, or interest to use e-cigarettes.
Second/third person experience	Reports someone else's use of e-cigarettes.
Personal opinion	Personal opinion related to e-cigarettes.
Marketing	Activities involved in the transfer of goods from the producer or seller to the consumer or buyer (eg, sales of e-cigarette products or accessories, job announcements, review of products posted by e-cigarette company/retailer).
Theme: Refers to the topical domain of the content in the tweet.	
Cessation	Mention of using e-cigarettes to quit smoking cigarettes or other non-e-cigarette tobacco products.
Health and safety	Direct or indirect reference to health consequences of e-cigarette use.
Underage usage	E-cigarette use by minors, especially high school age or under, (only if proof the person writing the tweet or mentioned in the tweet is underage).
Craving	Desire to use e-cigarettes (eg, “Stressful day. Time for my #vapepen”).
Other substances	E-cigarettes mentioned in association with other addictive substances, such as alcohol, caffeine.
Illicit substance use in e-cigarettes	Mention of using e-cigarettes for anything other than nicotine (eg, marijuana).
Policy or government	Mention of government or policy in relation to e-cigarettes including, regulation, deeming, bans, and restrictions.
Parental use of e-cigarettes	Tweet mentioning use of e-cigarettes by parents of the poster or parents of a person mentioned in the tweet.
Advertisement/ promotion	Ads for e-cigarettes, giveaways, samples, sales, direct links to seller’s websites, word-of-mouth, and reviews.
Flavors	Tweet discussing e-cigarette flavors (generic or mixed, including menthol).
