# Supplementary material for: Social Listening: A Content Analysis of E-Cigarette Discussions on Twitter
Source: J Med Internet Res. 2015 Oct 27;17(10):e243. doi: 10.2196/jmir.4969 (PMC4642379; doi:10.2196/jmir.4969)
Supplement: Multimedia Appendix 4 [file jmir_v17i10e243_app4.pdf]

Multimedia Appendix 4. Sample tweets by annotation category.

Category	Sample tweet
Relevance	
Relevant	Don't give me shit about my ecig mom. At least it's not a cigarette!!!
Not relevant	Today stats: 4 new followers and one new unfollower via [URL redacted][a]
Sentiment	
Positive	I love my Adidas shorts and my vape
Neutral	@[Name Redacted][a] E-cigs. Really hot topic. I'd be grateful to read your thoughts. Yea or nay?
Negative	E-cigarettes threaten to undo years of gains: Our view [URL redacted]via @usatoday
User descriptions	
Celebrity	From @BrunoMars: Day 1 E-Cig, Gotta do it!!! This is for you mom [URL redacted]
Government	From @US_FDA: FDA proposes new rule that would regulate additional tobacco products, e-cigarettes, pipe & waterpipe tobacco & more. [URL redacted]
Foundation/ organization	From @Campaign4Kids: Senators Call on FTC, FDA to Protect Consumers from False Advertising Claims by E-Cigarette Makers [URL redacted] via @SenatorBoxer
Reputable news source	From @Forbes: Chicago's e-cigarette ban has been blocked by "a surprise outpouring of opposition." [URL redacted]
Everyday person	From @[Name Redacted]: my girl just got me a new vape pen :)
E-cigarette community movement	From @E_Cigarette_: Smokeless Cigarettes More evidence e-cigs may help in quitting tobacco [URL redacted]
Retailers	From @SOURCEvapes: New #SOURCE #Orb attachments now available at TheVapeLifeStore/@VapeLifeWill! Don't miss out on the hottest new vape pen around! #newshit
Tobacco company	From @LIVcigs: FDA announced deeming regulations on #ecigs last week. They are wrought with ignorance and fear of…[URL redacted]
Bot/hacked	From @RMTSweeps: Entered to win a 2013 Kia Soul by shopping for e-cigs and e-liquid at www.MyFreedomSmokesCom Check it Out! [URL redacted]
Genre	
News/update	Marlboro maker Altria jumping into e-cigarettes as found on http://t.co/yUMvHtXFw6
Information	How an e-cig works. [URL redacted]

First person e-cigarette use or intent	Ecigs make me cough too much...
Second/third person experience	watching someone smoke an E-Cig is so bizarre.. like this dude is walking around the marvin center smoking something that looks like a pen
Personal opinion	If you smoke ecigs in school right next to me I have absolutely no respect for you..
Marketing	Best Value Beginners Complete Electronic Cigarette Starter Kit with E-Liquid - Cool Breeze E-Cigs Gr [URL redacted]
Theme	
Cessation	5 days out. Staying strong. Ecig has been helpful. #quitting [URL redacted]
Health/safety	Research has found that 3 out of 10 e-cigarettes contain two carcinogens that aren't necessarily found in regular cigarettes.
Underage use	[Name Redacted] just gave my 8 year old cousin a drag of her e-cig. #waytogo #auntoftheyear
Craving	I want my vape so bad right now.
Other substances	Vaping, drinking some macallans 18 and listening to some pink floyd. Life is good!!!
Illicit substance use in e-cigarettes	im vaping hash oil in my room and my parents r watching something w/ gunfire on tv so that xmas eve lol
Policy/ government	@[Name Redacted] #Ecigs are the most significant public health and technological breakthrough this century. Don't let the #FDA limit them.
Parental use of e-cigarettes	LOL I just found out my dad uses a vape HAHA
Advertisement/ promotion	I came, I saw, I vaped. Start vaping with VICE Vapes and let us be #yournewVICE [URL redacted]
Flavors	Have you tried our NEW Platinum Vanilla E-Liquid yet? Get more vape for your buck! [URL redacted]

[a]Twitter handles associated with individuals and URLs have been redacted to maintain user privacy.
