# Supplementary material for: Social Listening: A Content Analysis of E-Cigarette Discussions on Twitter
Source: J Med Internet Res. 2015 Oct 27;17(10):e243. doi: 10.2196/jmir.4969 (PMC4642379; doi:10.2196/jmir.4969)
Supplement: Multimedia Appendix 5 [file jmir_v17i10e243_app5.pdf]

Multimedia Appendix 5. Correlation matrix for content categories.

	Positive	Neutral	Negative	Celebrity	Government	Foundation	Reputable news	Everyday people	E-cigarette community	Retailer	Tobacco company	Bots\hacked	News	Information	Personal experience	2nd/3rd person experience	Personal opinion	Marketing	Cessation	Health/ safety	Underage usage	Craving	Other substances	Illicit substances	Policy/ government	Parental use	Advertisement/ promotion	Flavors
**Positive**	1																											
**Neutral**	0.7	1																										
**Negative**	0.58	0.17	1																									
**Celebrity**	0.03	0.02	0.02	1																								
**Government**	0.04	0.01	0.06	0	1																							
**Foundation**	0.13	0.08	0.09	0.01	0	1																						
**Reputable news**	0.1	0.09	0.04	0.01	0	0.01	1																					
**Everyday people**	0.21	0.13	0.15	0.09	0.04	0.15	0.12	1																				
**E-cigarette community**	0.17	0.11	0.11	0.03	0.01	0.05	0.04	0.59	1																			
**Retailer**	0.15	0.09	0.1	0.02	0.01	0.03	0.024	0.4	0.13	1																		
**Tobacco company**	0.07	0.04	0.05	0.01	0	0.02	0.01	0.19	0.06	0.04	1																	
**Bots/hacked**	0.06	0.03	0.05	0.02	0.01	0.03	0.02	0.37	0.12	0.08	0.04	1																
**News**	0.34	0.29	0.14	0.04	0.03	0.11	0.2	0.02	0.02	0.05	0.01	0	1															
**Information**	0.18	0.19	0.03	0.03	0.01	0.1	0.01	0.13	0.15	0.04	0.01	0.02	0.12	1														
**Personal experience**	0.2	0.11	0.14	0.01	0.01	0.06	0.04	0.3	0.16	0.14	0.07	0.11	0.15	0.21	1													
**2nd/3rd person experience**	0.16	0.11	0.1	0.01	0.01	0.03	0.02	0.15	0.09	0.05	0.03	0.06	0.09	0.12	0.15	1												
**Personal opinion**	0.02	0.09	0.13	0.02	0.01	0.05	0.05	0.08	0.09	0.11	0.06	0.09	0.19	0.26	0.32	0.18	1											
**Marketing**	0.31	0.22	0.19	0.02	0.01	0.04	0.04	0.35	0	0.36	0.16	0.24	0.15	0.21	0.26	0.15	0.32	1										
**Cessation**	0.05	0.04	0.02	0.01	0.02	0	0.03	0.01	0.01	0.01	0	0	0	0	0.02	0.02	0.07	0.05	1									
**Health/safety**	0.14	0.05	0.13	0.03	0.01	0.12	0.05	0.05	0.08	0.06	0.03	0.02	0.17	0.19	0.16	0.08	0.1	0.18	0.16	1								
**Underage usage**	0.18	0.06	0.18	0.02	0.03	0.11	0.03	0.06	0.05	0.05	0.02	0.03	0.14	0.02	0.03	0.03	0	0.11	0.03	0.09	1							
**Craving**	0.11	0.07	0.07	0.01	0.01	0.02	0.02	0.13	0.08	0.06	0.03	0.03	0.06	0.08	0.34	0.03	0.09	0.1	0.04	0.07	0.02	1						
**Other substances**	0.01	0	0.01	0.01	0	0	0.01	0.06	0.03	0.02	0.02	0.03	0.02	0.02	0.06	0	0.01	0.04	0.02	0	0.01	0.01	1					
**Illicit substances**	0.02	0.01	0.01	0	0	0.01	0.01	0.07	0.05	0.02	0.02	0.02	0.02	0.02	0.06	0.05	0.01	0.05	0.03	0.04	0.02	0	0.08	1				
**Policy/ government**	0.11	0.11	0.03	0.01	0.05	0.07	0.08	0.14	0.23	0.07	0.04	0.03	0.27	0.14	0.23	0.13	0.24	0.25	0	0.21	0.04	0.1	0.04	0.06	1			
**Parental use**	0.01	0.01	0	0.01	0	0.01	0.01	0.06	0.03	0.02	0.01	0.02	0.02	0.03	0.01	0.15	0.01	0.04	0	0.02	0.05	0.01	0	0	0.04	1		
**Advertisement/ promotion**	0.33	0.23	0.2	0.02	0.02	0.05	0.05	0.36	0.05	0.33	0.16	0.23	0.16	0.05	0.25	0.15	0.3	0.82	0.06	0.21	0.11	0.11	0.05	0.06	0.29	0.05	1	
**Flavors**	0.09	0.07	0.05	0.01	0.01	0.01	0.01	0.02	0.02	0.05	0.01	0	0.05	0.04	0.06	0.02	0.03	0.06	0.02	0.07	0.02	0.01	0.01	0.01	0.09	0	0.06	1
